# Feasibility and acceptability of a beverage intervention for Hispanic adults: a protocol for a pilot randomized controlled trial

**DOI:** 10.1186/s12937-018-0329-y

**Published:** 2018-02-09

**Authors:** Kristin E. Morrill, Benjamin Aceves, Luis A. Valdez, Cynthia A. Thomson, Iman A. Hakim, Melanie L. Bell, Jessica A. Martinez, David O. Garcia

**Affiliations:** 1 College of Agriculture & Life Sciences, Department of Nutritional Sciences, University of Arizona, Tucson, AZ USA; 2 Mel & Enid Zuckerman College of Public Health, Department of Health Promotion Sciences, University of Arizona, Tucson, AZ USA; 3 University of Arizona Cancer Center, Tucson, AZ USA; 4 Mel & Enid Zuckerman College of Public Health, Department of Epidemilogy and Biostatistics, University of Arizona, Tucson, AZ USA

**Keywords:** Green tea, Mediterranean lemonade, Sugar-sweetened beverages (SSBs), Hispanic

## Abstract

**Background:**

In the U.S., Hispanics have among the highest rates of overweight and obesity when compared to other racial/ethnic groups placing them at a greater risk for obesity-related disease. Identifying intervention strategies to reduce caloric intake and/or improve cardiometabolic health in Hispanics is critical to reducing morbidity and mortality among this large and growing population. Evidence exists to support diet-specific behavioral interventions, including beverage modifications, in reducing obesity-related health risks. However, the acceptability and feasibility of a beverage intervention in obese Hispanic adults has not been robustly evaluated.

**Methods:**

The objective of this pilot study is to assess the feasibility and acceptability of a randomized, controlled beverage intervention in 50 obese Hispanic adults ages 18–64 over 8-weeks. Eligible participants were obese (30–50.0 kg/m^2^), between the ages 18–64, self-identified as Hispanic, and were able to speak, read, and write in either English and/or Spanish. Study recruitment was completed August 2017. Upon the completion of baseline assessments, participants will be randomized to either Mediterranean lemonade, Green Tea, or flavored water control. After completing a 2-week washout period, participants will be asked to consume 32 oz. per day of study beverage for 6-weeks while avoiding all other sources of tea, lemonade, citrus, juice, and other sweetened beverages; water is permissible. Primary outcomes will be recruitment, retention, and acceptability of the intervention strategies. Our study will also evaluate participant-reported tolerance and as an exploratory aim, assess safety/toxicity-related to renal and/or liver function. Fasting blood samples will be collected at baseline and 8-weeks to assess the primary efficacy outcomes: total cholesterol, high-density lipoprotein (HDL), and low-density lipoprotein (LDL). Secondary outcomes include fasting glucose, hemoglobin A1c (HbA1c), and high-sensitivity C-reactive protein (hs-CRP).

**Discussion:**

This pilot study will provide important feasibility, safety, and early efficacy data necessary to design a larger, adequately-powered randomized controlled trial.

**Trial registration:**

NCT02911753 (ClinicalTrials.gov). Registered September 19, 2016. Last updated November 1, 2017.

## Background

Approximately 40% of Hispanic men in the U.S. are obese (body mass index, BMI ≥ 30.0 kg/m^2^) compared to 32% of non-Hispanic white men and 37% of non-Hispanic black men [1]. Among women, rates of obesity remain high for Hispanics at 43% compared to only 34% of non-Hispanic women [1]. Obesity is linked to cardiovascular disease, metabolic syndrome, type 2 diabetes, hypertension, dyslipidemia, osteoarthritis, sleep apnea, gallstones, and certain forms of cancer [2, 3]. Risk of developing these comorbid conditions is positively associated with increasing BMI. Consequently, Hispanic adults, regardless of gender, have a higher prevalence of certain obesity-related comorbidities in the U.S. relative to other racial/ethnic subgroups. For example, recent estimates suggest that Hispanic adults are 1.4 times more likely than non-Hispanic white adults to develop diabetes [4]. Additionally, cardiometabolic abnormalities such as hypercholesterolemia, hypertriglyceridemia, and hyperglycemia are more prevalent in this population subgroup overall [5, 6]. Evidence suggests that obese individuals tend to have above normal lipid status and this is associated with a 3-fold higher risk for heart attack [7], placing obese Hispanics at further risk. Identifying intervention strategies to reduce caloric intake and/or improve cardiometabolic health in Hispanics is critical to reducing morbidity and mortality among this large and growing population [8, 9].

### Beverage consumption

Consumption of energy-dense beverages is high in the U.S., where sugar-sweetened beverages (SSBs) are the primary source of added sugar calories and a contributing factor of obesity. Recent trends in beverage consumption in the U.S. indicate SSB consumption is declining overall, however, levels of consumption remain highest among Mexican Americans, non-Mexican Hispanics, and non-Hispanic blacks [10]. Consumption of SSBs also vary by race/ethnicity in Arizona with 39% of Hispanics consuming SSBs ≥1 time/day compared to 21.1% of non-Hispanic whites and 32.9% non-Hispanic blacks [11]. Associations between SSB consumption and obesity-related metabolic disturbances have been investigated [12–14] and whether or not reducing SSB consumption may reduce the prevalence of obesity and obesity-related diseases has previously been the subject of debate [15, 16]. While notable efforts have been made in developing interventions to reduce SSB consumption in children and adolescents [17, 18], less attention has been placed on beverage interventions in adults. In a recent systematic review and meta-analysis by Vargas-Garcia et al. [19], investigators sought to evaluate the effectiveness of interventions aimed at reducing SSB or increasing water consumption. Of the 40 studies identified in the review, only 11 included adult participants. Despite this, results from interventions aimed at reducing SSB intake or increasing water intake in adults are promising. For instance, Zoellner et al. [20] examined the effectiveness of a behavioral and health literacy intervention for adults targeting SSB consumption (SIPsmartER) compared to an intervention targeting physical activity (MoveMore). Significant decreases in SSB calories and BMI were observed in the SIPsmartER group compared to the MoveMore group over a period of 6-months, however, only 1% of participants were Hispanic. Similarly, Rodriguez-Ramirez et al. [21] examined the effects of reducing SSB consumption over sustained periods of time in a group of 268 overweight Mexican women and found that women who received a water and nutrition education (where water was home-delivered during the intervention period) significantly improved the quality of their diets when compared to women who received only the nutrition education [21]. This included significant reductions in SSB consumption, suggesting that encouraging water consumption may be an effective strategy in reducing consumption of SSBs, however, more research is needed.

### Alternate beverages

Similarly, our study seeks to replace dietary SSBs with healthier alternatives while simultaneously modifying cardiometabolic measures with the bioactive dietary constituents found in two beverages. Specifically, our group and others have shown improvements in metabolic activity for both green tea [22, 23] and Mediterranean lemonade [24–26]. The health-promoting bioactivity due to the consumption of green tea and Mediterranean lemonade is thought to be in relation to the exposure to compounds known to regulate biological pathways such as green tea polyphenols [27] and citrus-derived limonene [24], as well as the opportunity to replace high-glycemic response beverages such as sweetened sodas and juice products [28, 29].

Specifically, researchers have found that green tea consumption over extended periods of time is associated with decreased body weight and lower low-density lipoprotein (LDL) levels, particularly among participants who are at risk for cardiovascular disease [22, 27]. Further, favorable trends of increased blood high-density lipoprotein (HDL) have been associated with consumption of green tea [27]. Mediterranean lemonade, a beverage consisting of two full lemons with peels and water, is a low-calorie beverage alternative to energy-dense SSBs with potential health-promoting effects [24–26]. It has been previously identified as a source of *d-*limonene, a bioactive compound found in high concentrations in citrus peel [26]. Its potential anti-proliferative effects, bioavailability, proposed physiological mechanisms, and safety have been reviewed by our group elsewhere [24].

## Aims and study hypotheses

This pilot study will expand on earlier research by our team using beverage interventions to enhance cardiometabolic health. This study’s primary objective is to assess the feasibility and acceptability of a beverage intervention in obese Hispanic adults. Primary feasibility outcomes will be recruitment, retention, and acceptability. Beverage intake will also be assessed through intake diaries and beverage return rates during weekly clinic visits. The preliminary efficacy of the beverage intervention will assess changes in total cholesterol, HDL, and LDL over 8-weeks. Secondary outcomes are changes in fasting glucose, hemoglobin A1c (HbA1c), and high-sensitivity C-reactive protein (hs-CRP). Our study will evaluate participant-reported tolerance and as an exploratory aim, our study will also assess safety/toxicity-related to renal and/or liver function as albeit limited, evidence from green tea polyphenol E supplementation studies have reported a few cases of hepatic toxicity with on-going supplementation [30]. Renal and hepatic toxicity function will be assessed at baseline and 8-weeks. The aforementioned outcomes will be examined to test the following hypotheses: a) recruitment and retention of obese Hispanic participants in an 8-week beverage intervention study is feasible; b) the consumption of green tea and Mediterranean lemonade will be well-tolerated with high adherence; and c) green tea and Mediterranean lemonade intake will result in an improved cardiometabolic profile at the end of 8-weeks.

## Methods/Design

### Design

**Fig. 1:**
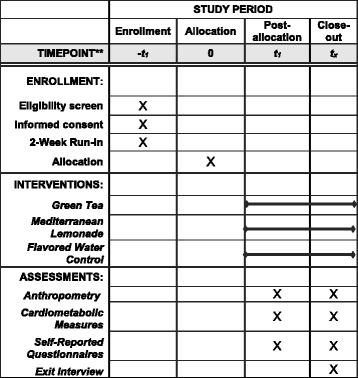
Content for the schedule of enrollment, interventions, and assessments according to SPIRIT requirements

This study is a pilot randomized controlled trial where participants are randomized to one of three beverages: Green Tea (GT), Mediterranean lemonade (ML), or a Flavored Water control (FW). Participants will be stratified by gender using block randomization and an allocation ratio of 2:2:1 with variable block sizes. A computer randomization system will be used to complete the randomization assignment. We proposed to consent 150 individuals to randomize 50 participants into our 8-week study. Following consent and baseline assessments, randomization assignment will be determined and documented in the participants’ study record and shared with the participant. Assessments will be performed by blinded study personnel. Under no circumstances will unblinding be permissible. Investigators, intervention staff, and participants will not be blinded for the study. The schedule of the enrollment, interventions, and assessments according to SPIRIT requirements is shown in Fig. 1.

### Setting of the human research

Research activities will take place at the University of Arizona Collaboratory for Metabolic Disease Prevention and Treatment in Tucson, AZ. All procedures have been approved by the University of Arizona Institutional Review Board.

### Study population

We recruited 50 obese Hispanic adults aged 18–64 years living in the Tucson area. Individuals were considered eligible if they met all of the following criteria: self-identified as Hispanic, 18–64 years of age, BMI between 30 and 50.0 kg/m^2^, able to provide informed consent, and able to speak, read, and write in either English and/or Spanish. Individuals were excluded if they met any of the following criteria: diagnosis of diabetes mellitus; history of liver disease; current medication for glucose control; cholesterol control; uncontrolled blood pressure; current eating disorders such as anorexia nervosa; bulimia, etc. (likely to make adherence to prescribed beverage intake difficult); current alcohol or substance abuse; currently treated for psychological issues (i.e. depression, bipolar disorder, etc.); taking psychotropic medications within the previous 12 months or hospitalized for depression within the previous 5 years; report exercise on ≥3 days per week for ≥20 min per day over the past 3 months; reported weight loss of ≥5% or participating in a weight reduction diet program in the past 3 months; reported plans to relocate to a location that limits their access to the study site or having employment, personal, or travel commitments that prohibit attendance to all of the scheduled assessments; reported plans to relocate to a location that limits their access to the study site or having employment, personal, or travel commitments that prohibit attendance to all of the scheduled assessments; or reported consumption of ≥1 cup of green tea and/or citrus fruit daily and were not willing to complete 2-week run-in period.

### Study recruitment

Recruitment efforts primarily targeted local community-based settings such as clinics, health fairs, and outdoor marketplaces frequented by Hispanic communities in Tucson, AZ. Additional recruitment strategies included the use of social media posts (e.g., Facebook, and Craigslist) and health provider initiated approaches (e.g., patient referral). Participants of preliminary studies who had expressed interest in health behavior research and had signed consent to be contacted for future intervention studies also were contacted. Potential participants were instructed to call study staff and a telephone screening was conducted to determine initial eligibility. Interested individuals engaged at recruitment settings were given the option of being screened on-site or during a future phone call with study staff. Telephone and on-site screenings included a detailed description of the study and its potential risks and benefits. Upon the participants’ verbal agreement, study staff asked questions regarding medical history and other pertinent questions related to exclusion/inclusion criteria. Notably, recruitment was completed in August 2017, however, baseline assessments for the study are still underway.

### Informed consent

All eligible participants will be invited to the Collaboratory where complete details of the study will be provided in the participants’ preferred language (Spanish or English). During this time, participants will be encouraged to ask questions about the study’s procedures. Interested participants will provide written informed consent (see appendices) to study personnel using consent forms that are available in the participants’ preferred language.

### Certificate of confidentiality

To help us protect the participant’s privacy, we obtained a Certificate of Confidentiality (CC-DK-17-003) from the National Institutes of Health, National Institute of Diabetes and Digestive and Kidney Diseases (NIDDK). This Certificate can be used legally to refuse to disclose information that may identify participants in any federal, state, or local civil, criminal, administrative, legislative, or other proceedings, for example, if there is a court subpoena.

### Study intervention

Study personnel will prepare all study beverages. Lipton® Decaffeinated Green tea will be prepared using a ratio of 4 tea bags per 32 oz. water. Upon reaching a boil, tea bags will be steeped into water for 3–5 min. When slightly cooled, 4 drops of Liquid No Calorie Stevia™ will be added per 32 oz. of tea. To prepare the Mediterranean lemonade, two full lemons will be de-seeded and blended with 32 oz. of water and 3 drops of Liquid No Calorie Stevia™. The control beverage will be prepared using 3 drops of Crystal Light Liquid Drink Mix (strawberry lemonade) per 32 oz. of water. All beverages will be prepared 2 days prior to participant pick-ups. All green tea will be kept frozen while the Mediterranean lemonade and flavored water will be refrigerated for storage and distribution to study participants. Preparation and storage procedures were informed by prior chemical analysis of bioactive components in the beverages across 7 days to assure optimal exposure. Mean epigallocatechin gallate (EGCG), the main polyphenol found in green tea, was 161.5 mg per 32 oz. and mean *d-*limonene was 373.4 μg/ml per 32 oz. in the Mediterranean lemonade. All analyses were conducted by the UA Cancer Center Analytical Chemistry Shared Resource. This information, as well as nutrient information, is displayed in Table 1.

**Table 1:** Nutrient and bioactive components information of study beverages

	Kcal	Carbs (g)	Protein (g)	Fat (g)	Vitamin C (mg)	Fiber (g)	Sugar (g)	EGCG^a^ (mg)	*d*-limonene^b^ (μg/ml)
ML	37	11.64	1.28	0.35	61.48	3.25	3.73	–	373.4
GT	13	0.83	2.08	0	2.84	0	0.83	161.5	–
FW	7	2.76	0.01	0	0	0	0	–	–

*ML* Mediterranean lemonade, *GT* green tea, *FW* flavored water, *EGCG* epigallocatechin gallate. Nutrient information represents amount per 32 oz of beverage. Retrieved from Nutrition Data System for Research (NDSR) 2017

^a^Data are presented as the mean EGCG per 32 oz of GT, analyzed over a 7 day period

^b^Data are presented as the mean *d*-limonene per 32 oz of ML, analyzed over a 7 day period

Participants will complete a 2-week run-in period prior to beginning the 6-week intervention. During this period, participants will be asked to stop all tea and citrus fruit consumption and limit consumption of any beverages except water. Each participant will be provided with a 32-oz Hydro Flask® to support regular intake of the prescribed beverage. This run-in period will determine the feasibility of consuming 32 oz. each day for participant adherence to the beverage intervention.

Upon successful completion of baseline assessments and 2-week run-in, participants will be randomized to a beverage assignment. All participants will be advised to consume the entire beverage assigned on a daily basis (rather than save up and consume large amounts on fewer days). At the start of the intervention, participants will be asked to continue avoiding all other sources of tea, lemonade, and citrus, limit coffee consumption to 2 cups/day, avoid all sweetened beverages other than study-provided drinks, consume water ad lib, and avoid alcohol in excess of 1 drink/day for women and 2 drinks/day for men. In addition, participants will be asked to self-monitor their beverage consumption behaviors using a weekly journal to help habitually regulate beverage intake. Participants will be responsible for picking up 1 week’s worth of beverages once per week from the study clinic. Participants will be asked to return any un-consumed beverages during weekly pick-ups; study staff will measure and record any amount not consumed. Weekly beverage journals will also be collected and new ones distributed for use the following week. Brief in-person interviews or “check-ins” lasting 5–10 min will also take place during this time with bilingual study staff. These interviews will follow a script to review beverage-related behaviors and identify and address specific barriers for adherence to study beverage intake.

### Retention strategies

We will use common strategies to enhance participant retention, including: 1) collecting contact information of participants and at least two family members; 2) program reminders; 3) incentives to complete assessments; and 4) contacting participants at their preferred time by their preferred method (i.e. call or text) in their preferred language.

### Participant-reported tolerance or toxicities

Potential risks may include but are not limited to: nausea, vomiting, frequent bowel movements, flatulence (gas), acid reflux, excess burping, heartburn, and bloating. If a participant experiences any of these signs/symptoms associated with beverage intake, they will be given the option to withdraw from the study or change to the alternate beverage intervention (but not flavored water) after a 1-week washout period. Participants will be asked on a weekly basis about any issues related to their beverage consumption and this information will be noted. Prior literature has reported a few cases of hepatic toxicity related to supplementation with high-dose green tea polyphenols [30]. No evidence exists to suggest toxicity or safety issues related to the green tea nor Mediterranean lemonade beverages consumed in the amounts prescribed for this study. Therefore, renal and hepatic function will be monitored at study beginning and end using alanine transaminase (ALT) or aspartate transaminase (AST) values. Abnormal values will be shared with the participant and the participant will be advised to visit their physician for evaluation. In the event this occurs, participants will not receive additional compensation and the study will not cover additional costs for physician follow-up.

## Methods for assessing study outcomes and potential confounders

### Anthropometry

Height, body weight, waist circumference, and resting blood pressure will be measured at baseline and 8-weeks. Height will be measured to the nearest 0.1 cm (cm) using a ShorrBoard® wall-mounted stadiometer with participants removing their shoes prior to the measurement. Two measurements will be taken. A third measurement will be taken if the first two measurements differ by more than 0.5 cm. The average of the two measurements which met the criteria above will be recorded for data collection. Body weight will be measured using a Seca 876 scale to the nearest 0.1 kg (kg) on a digital scale without shoes. Two measurements will be taken. A third measurement will be taken if the first two measurements differ by more than 0.2 kg. The average of the two measurements which meets the criteria above will be recorded for data collection. BMI will be calculated using body weight in kilograms divided by squared height in meters (kg/m^2^). Waist circumference will be obtained using a Gulick measuring tape recorded to the nearest 0.1 cm. Waist circumference will be measured in the horizontal plane directly at the umbilicus. Two measurements will be taken at each site. A third measurement will be taken if the first two measurements differ by more than 2.0 cm. The average of the two measurements closest to each other will be recorded for data collection.

Resting blood pressure and heart rate will be measured using the Omron Digital Blood Pressure Monitor HEM-907XL on the participant’s left arm. Using a Gulick measuring tape, an arm measurement will be performed on the lateral aspect of the left arm at the midpoint between the acromion process to the olecranon process to determine the appropriate cuff size. Upon a five-minute resting period with the participant in an upright position with feet flat on the floor, two blood pressure measurements will be taken with a one-minute time period between each measurement. A third blood pressure will be taken if the mean difference between the systolic blood pressure measurements differs by 10 mmHg or greater and/or the diastolic blood pressure measurements differs by 6 mmHg or greater. The average of the two measurements which meets the criteria above will be recorded for data collection. In addition, if the mean resting systolic blood pressure is ≥150 mmHg or average diastolic blood pressure is ≥100 mmHg at baseline, the participant will be excluded from participation and referred back to their physician.

### Biological samples

Emerging evidence indicates that genetic variation may impact the efficacy of lifestyle behavioral interventions [31]. Therefore, we will perform a buccal cell collection by using a cytology brush to brush the inside of each cheek for 30 s for the extraction of DNA and subsequent genetic analyses. This *optional* non-invasive, self-collection of buccal cells will occur at one time and will be performed at either the baseline (preferable) or follow-up visit. Participants also will have the option of allowing the study investigators to store some of the blood that was taken but not used for other tests. This will allow the researchers to derive metabolomics and molecular data that are complementary in obesity research and provide value for enhancing precision care in this health disparate population.

### Cardiometabolic measures

Fasting blood samples (venipuncture; 25 mL), will be collected at baseline and 8-weeks, by a trained phlebotomist, for the purpose of examining the following cardiometabolic measures: total cholesterol, triglycerides, HDL, LDL, very low-density lipoprotein (VLDL), HbA1C, hs-CRP, and fasting glucose. A comprehensive metabolic liver/kidney panel will be performed to assess the following: albumin, globulin, A/G ratio, total protein, alkaline phosphatase, ALT, AST, total bilirubin, blood urea nitrogen (BUN), calcium, creatinine, BUN/creatinine ratio, glomerular filtration rate/estimated, and electrolytes (sodium, potassium, chloride, carbon dioxide). All laboratory tests will be performed by an independent, clinical lab (Banner University Medical Center, Tucson, AZ), blinded to the study outcomes.

### Physical activity

Physical activity will be assessed at baseline and 8-weeks using the Global PA Questionnaire (GPAQ) [32, 33]. It is available in both English and Spanish and provides minutes/week of physical activity of varying intensity and type. The GPAQ has been validated compared to accelerometer data in determining time spent in moderate-to-vigorous physical activity (MVPA) and to assess changes in physical activity over time. Cleland et al. [33] found a moderate agreement between the GPAQ and accelerometer for MVPA mins/day (*r* = 0.48) and results for agreement in changer over time showed moderate correlation (*r* = 0.52, *p* = 0.12). Similar correlations have been reported for the GPAQ compared to accelerometer data for Hispanic women participating in a 6-month physical activity intervention [32].

### Diet

Diet assessment will be completed at baseline and 8-weeks using the Southwestern Food Frequency Questionnaire (SWFFQ) [34–36], a bilingual FFQ that includes food items commonly consumed by Mexican Americans and uses Mexican names for food items commonly given different names by other Spanish speakers (e.g., “naranja”, not “china”, for “orange”). Output data from the SWFFQ will allow us to calculate daily intake of total sugar, saturated fat, sodium, fiber, whole grains, and fruits/vegetables. Internal validity of the SWFFQ compares favorably with 24-h recall (*r* = 0.82) [34]. SWFFQ data will be analyzed using the 2009 United States Department of Agriculture (USDA) Nutrient Data Bank.

### Tea consumption

We will assess tea consumption at baseline and 8-weeks using the Arizona Tea Questionnaire. This 28-item scannable questionnaire has been tested for short and long-term reliability as well as relative validity [37]. This questionnaire asks about usual tea intake over the past year, as well as lifetime consumption patterns including amount, type, and preparation technique. The output is provided in total flavonoids, total polyphenols, catechins, theaflavins, thearubigins, caffeine, and gallic acid.

### Psychosocial measures and acculturation

We will use the following self-reported questionnaires to assess psychosocial measures at baseline and 8-weeks which may influence diet and physical activity behaviors: Self-efficacy [38] and social support for diet [39], The Acculturation Rating Scale for Mexican Americans–II (ARSMA-II) [40] will be used to measure acculturation related to language, ethnic identity, and ethnic interaction. The reliability, and validity of the ARSMA-II are well established in English and Spanish [40].

## Statistical analysis plan

### Feasibility outcomes

The primary feasibility outcomes are recruitment and retention. We aimed to recruit, on average, approximately 2–3 participants per week during the active recruitment phase. A recruitment rate of less than this would indicate a lack of feasibility. We recorded the number of Hispanic adults who contacted the researchers and expressed interest in participation, the number screened for eligibility, and the number ineligible for study inclusion and the reason for their ineligibility. Retention will be assessed by calculating the proportion of participants who completed the study out of the number enrolled, with a 95% confidence interval.

### Treatment satisfaction/acceptability

At the completion of the study, participants will take part in an exit interview where they will be asked to rate their overall satisfaction with the intervention, if they would consider a longer-term beverage intervention, and finally, if they would recommend the program to others. Participants will also be asked questions regarding satisfaction with their overall progress and for changing dietary beverage habits. Each item will be rated on a Likert scale with higher scores indicating greater program favorability. Open-ended questions will be used to seek participant input on modifications that could be made to improve acceptability and effectiveness of the intervention. The responses will be used to identify which recruitment and intervention components were well received, which could be improved, and which were not acceptable.

### Efficacy outcomes

Descriptive statistics, including means, ranges, and standard deviations, will be calculated for the primary efficacy outcomes, cholesterol/lipid levels, and the secondary outcomes: fasting glucose, HbA1c, and hs-CRP. Mixed models will be used to compare arms in each of these outcomes’ change from baseline. We will include fixed effects of intervention arm, time and their interaction to allow for different patterns of change between the arms, as well as a random participant effect to account for the longitudinal nature of the data. Baseline values of the outcomes will be included in the dependent variable vector. The changes from baseline, and the differences between arms in change from baseline will be estimated using contrasts from the mixed models. Mixed models yield unbiased estimates for data that are missing completely at random (when missingness does not depend on any observed or unobserved data) and missing at random (when missingness may depend on observed data, such as the baseline value of the outcomes), as these models perform an implicit imputation [41, 42]. Data from all patients who are randomized will be analyzed in the arm that they were randomized to.

### Sample size calculation

The total sample size of 50 will provide 95% confidence intervals for the primary feasibility outcomes (proportion of eligible participants who enroll and the proportion of participants retained in the study) that are no wider than 0.28 (±margin of error = 0.14), conservatively assuming a base proportion of 0.5, which maximizes the standard error. The trial will be considered feasible if the proportion retained is greater than 70%. If retention in the study is 81% or greater, the 95% confidence interval will not contain this 70% cutoff. Power will be insufficient to detect a minimum important difference of a 6 mg/dL change in fasting lipids from baseline, the primary efficacy outcome, based on variance estimates from a trial using a different fruit [43] (standard deviation ≈25 mg/dL). We acknowledge that we are not sufficiently powered to detect important differences between arms, but if the 95% confidence intervals for the comparison of the arms contain important differences we will take this as indication of a promising intervention. Variance components estimated from this study will be used to design an adequately powered trial.

### Data management

Data management will be conducted according to Good Clinical Practice guidelines to enhance data quality assurance and control. This will include standard operating procedures and staff trainings for data acquisition, entry, and processing. Further, information obtained from this research will be kept confidential to respect the participant’s privacy. All records will be stored and locked in a file cabinet. In addition, all research databases will have password-controlled access, and the researchers will control this access. Data analyses will take place using a dedicated study computer upon study completion. No interim analyses will be performed. Study data will be uniquely coded for each subject and then entered via secure server to a Research Electronic Data Capture (REDCap) database maintained by the Clinical and Translational Sciences Research Center (CATS) at the University of Arizona. Only local study personnel will have access to identified study data. Once the study is complete the study records will be transferred to the University of Arizona storage facility and kept in accordance with applicable laws. All participant data (including signed informed consent) will be stored for 6 years after the completion of the project. Results from this study will be disseminated through peer-reviewed manuscripts and presentations at national meetings. Participants will be provided with a lay summary of the research findings and the peer-reviewed manuscripts.

## Discussion

Hispanics report high intake of SSBs and are significantly underrepresented in research relative to the extent in which they are impacted by obesity and metabolic abnormalities. Our work serves to address this critical gap. Further, this pilot study will provide important feasibility, safety, and early efficacy data necessary to design a larger, adequately-powered randomized controlled trial.

## Abbreviations

ALT: Alantine transaminase
ARSMA-II: Acculturation Rating Scale for Mexican Americans – II
AST: Aspartate transaminase
BMI: Body mass index
BUN: Blood urea nitrogen
CATS: Clinical and Translational Sciences Research Center
CDC: Center of Disease Control
EGCG: Epigallocatechin gallate
GPAQ: Global Physical Activity Questionnaire
HbA1c: Hemoglobin A1c
HDL: High-density lipoprotein
hs-CRP: High-sensitivity C-reactive protein
LDL: Low-density lipoprotein
MVPA: Moderate-to-vigorous physical activity
REDCap: Research Electronic Data Capture
SSBs: Sugar-sweetened beverages
SWFFQ: Southwestern Food Frequency Questionnaire
USDA: United States Department of Agriculture
VLDL: Very low-density lipoprotein

## References

[1] Ogden CL, Carroll MD, Kit BK, Flegal KM (2014). Prevalence of childhood and adult obesity in the United States, 2011-2012. JAMA.

[2] Pi-Sunyer FX (2002). The obesity epidemic: pathophysiology and consequences of obesity. Obes Res.

[3] Klein S, Burke LE, Bray GA, Blair S, Allison DB, Pi-Sunyer X, Hong Y, Eckel RH (2004). Clinical implications of obesity with specific focus on cardiovascular disease: a statement for professionals from the American Heart Association Council on nutrition, physical activity, and metabolism: endorsed by the American College of Cardiology Foundation. Circulation.

[4] Schiller JS, Lucas JW, Ward BW, Peregoy JA (2012). Summary health statistics for U.S. adults: National Health Interview Survey, 2010. Vital Health Stat.

[5] Go AS, Mozaffarian D, Roger VL, Benjamin EJ, Berry JD, Blaha MJ, Dai S, Ford ES, Fox CS, Franco S (2014). Heart disease and stroke statistics--2014 update: a report from the American Heart Association. Circulation.

[6] Daviglus ML, Pirzada A, Talavera GA (2014). Cardiovascular disease risk factors in the Hispanic/Latino population: lessons from the Hispanic community health study/study of Latinos (HCHS/SOL). Prog Cardiovasc Dis.

[7] Hubert HB, Feinleib M, McNamara PM, Castelli WP (1983). Obesity as an independent risk factor for cardiovascular disease: a 26-year follow-up of participants in the Framingham heart study. Circulation.

[8] Lindberg NM, Stevens VJ, Halperin RO (2013). Weight-loss interventions for Hispanic populations: the role of culture. J Obes.

[9] Ceballos NA. Weight loss interventions in the Mexican American Community. In: Brennan VM, Kumanyika SK, Zambrana RE, editors. Obesity interventions in underserved communities: Evidence and directions. Baltimore: Johns Hopkins University Press; 2014. p. 123-51.

[10] Bleich SN, Vercammen KA, Koma JW, Li Z. Trends in Beverage Consumption Among Children and Adults, 2003-2014. Obesity (Silver Spring) 2018;26(2):432-41. doi: 10.1002/oby.22056. 10.1002/oby.22056 29134763

[11] Park S, Xu F, Town M, Blanck HM (2016). Prevalence of sugar-sweetened beverage intake among adults--23 states and the District of Columbia, 2013. MMWR Morb Mortal Wkly Rep.

[12] Vartanian LR, Schwartz MB, Brownell KD (2007). Effects of soft drink consumption on nutrition and health: a systematic review and meta-analysis. Am J Public Health.

[13] Ma J, Fox CS, Jacques PF, Speliotes EK, Hoffmann U, Smith CE, Saltzman E, McKeown NM (2015). Sugar-sweetened beverage, diet soda, and fatty liver disease in the Framingham heart study cohorts. J Hepatol.

[14] Dhingra R, Sullivan L, Jacques PF, Wang TJ, Fox CS, Meigs JB, D'Agostino RB, Gaziano JM, Vasan RS (2007). Soft drink consumption and risk of developing cardiometabolic risk factors and the metabolic syndrome in middle-aged adults in the community. Circulation.

[15] Kaiser KA, Shikany JM, Keating KD, Allison DB (2013). Will reducing sugar-sweetened beverage consumption reduce obesity? Evidence supporting conjecture is strong, but evidence when testing effect is weak. Obes Rev.

[16] Hu FB (2013). Resolved: there is sufficient scientific evidence that decreasing sugar-sweetened beverage consumption will reduce the prevalence of obesity and obesity-related diseases. Obes Rev.

[17] de Ruyter JC, Olthof MR, Seidell JC, Katan MB (2012). A trial of sugar-free or sugar-sweetened beverages and body weight in children. N Engl J Med.

[18] Ebbeling CB, Feldman HA, Chomitz VR, Antonelli TA, Gortmaker SL, Osganian SK, Ludwig DS (2012). A randomized trial of sugar-sweetened beverages and adolescent body weight. N Engl J Med.

[19] Vargas-Garcia EJ, Evans CEL, Prestwich A, Sykes-Muskett BJ, Hooson J, Cade JE (2017). Interventions to reduce consumption of sugar-sweetened beverages or increase water intake: evidence from a systematic review and meta-analysis. Obes Rev.

[20] Zoellner JM, Hedrick VE, You W, Chen Y, Davy BM, Porter KJ, Bailey A, Lane H, Alexander R, Estabrooks PA (2016). Effects of a behavioral and health literacy intervention to reduce sugar-sweetened beverages: a randomized-controlled trial. Int J Behav Nutr Phys Act.

[21] Rodriguez-Ramirez S, Gonzalez de Cosio T, Mendez MA, Tucker KL, Mendez-Ramirez I, Hernandez-Cordero S, Popkin BM (2015). A water and education provision intervention modifies the diet in overweight Mexican women in a randomized controlled trial. J Nutr.

[22] Stendell-Hollis NR, Thomson CA, Thompson PA, Bea JW, Cussler EC, Hakim IA (2010). Green tea improves metabolic biomarkers, not weight or body composition: a pilot study in overweight breast cancer survivors. J Hum Nutr Diet.

[23] Baladia E, Basulto J, Manera M, Martinez R, Calbet D (2014). Effect of green tea or green tea extract consumption on body weight and body composition; systematic review and meta-analysis. Nutr Hosp.

[24] Miller JA, Thompson PA, Hakim IA, Chow H-HS, Thomson CA (2011). D-limonene: a bioactive food component from citrus and evidence for a potential role in breast cancer prevention and treatment. Oncol Rev.

[25] Miller JA, Lang JE, Ley M, Nagle R, Hsu CH, Thompson PA, Cordova C, Waer A, Chow HH (2013). Human breast tissue disposition and bioactivity of limonene in women with early-stage breast cancer. Cancer Prev Res (Phila).

[26] Miller JA, Hakim IA, Chew W, Thompson P, Thomson CA, Chow HH (2010). Adipose tissue accumulation of d-limonene with the consumption of a lemonade preparation rich in d-limonene content. Nutr Cancer.

[27] Zheng XX, Xu YL, Li SH, Liu XX, Hui R, Huang XH (2011). Green tea intake lowers fasting serum total and LDL cholesterol in adults: a meta-analysis of 14 randomized controlled trials. Am J Clin Nutr.

[28] Sae-tan S, Grove KA, Lambert JD (2011). Weight control and prevention of metabolic syndrome by green tea. Pharmacol Res.

[29] Kao YH, Hiipakka RA, Liao S (2000). Modulation of obesity by a green tea catechin. Am J Clin Nutr.

[30] Isomura T, Suzuki S, Origasa H, Hosono A, Suzuki M, Sawada T, Terao S, Muto Y, Koga T (2016). Liver-related safety assessment of green tea extracts in humans: a systematic review of randomized controlled trials. Eur J Clin Nutr.

[31] Bray MS, Loos RJF, McCaffery JM, Ling C, Franks PW, Weinstock GM, Snyder MP, Vassy JL, Agurs-Collins T, The Conference Working G (2016). NIH working group report—using genomic information to guide weight management: from universal to precision treatment. Obesity.

[32] Hoos T, Espinoza N, Marshall S, Arredondo EM (2012). Validity of the global physical activity questionnaire (GPAQ) in adult Latinas. J Phys Act Health.

[33] Cleland CL, Hunter RF, Kee F, Cupples ME, Sallis JF, Tully MA (2014). Validity of the global physical activity questionnaire (GPAQ) in assessing levels and change in moderate-vigorous physical activity and sedentary behaviour. BMC Public Health.

[34] Taren DL, Tobar M, Ritenbaugh C, Graver E, Whitacre R, Aickin M (2000). Evaluation of the southwest food frequency questionnaire. Ecol Food Nutr.

[35] Martinez ME, Marshall JR, Graver E, Whitacre RC, Woolf K, Ritenbaugh C, Alberts DS (1999). Reliability and validity of a self-administered food frequency questionnaire in a chemoprevention trial of adenoma recurrence. Cancer Epidemiol Biomark Prev.

[36] Garcia R, Taren DL, Teufel N (1999). Factors associated with the reproducibility of specific food items from the southwestern food frequency questionnaire. Ecol Food Nutr.

[37] Hakim IA, Hartz V, Harris RB, Balentine D, Weisgerber UM, Graver E, Whitacre R, Alberts D (2001). Reproducibility and relative validity of a questionnaire to assess intake of black tea polyphenols in epidemiological studies. Cancer Epidemiol Biomark Prev.

[38] Sallis JF, Pinski RB, Grossman RM, Patterson TL, Nader PR (1988). The development of self-efficacy scales for healthrelated diet and exercise behaviors. Health Educ Res.

[39] Sallis JF, Grossman RM, Pinski RB, Patterson TL, Nader PR (1987). The development of scales to measure social support for diet and exercise behaviors. Prev Med.

[40] Cuellar I, Arnold B, Maldonado R (1995). Acculturation rating scale for Mexican Americans-II: a revision of the original ARSMA scale. Hisp J Behav Sci.

[41] Bell ML, Fairclough DL (2014). Practical and statistical issues in missing data for longitudinal patient-reported outcomes. Stat Methods Med Res.

[42] Molenberghs G, Thijs H, Jansen I, Beunckens C, Kenward MG, Mallinckrodt C, Carroll RJ (2004). Analyzing incomplete longitudinal clinical trial data. Biostatistics.

[43] Dow CA, Going SB, Chow HH, Patil BS, Thomson CA (2012). The effects of daily consumption of grapefruit on body weight, lipids, and blood pressure in healthy, overweight adults. Metabolism.

